# Is There a Role for Hyperbaric Oxygen Therapy in Reducing Long-Term COVID-19 Sequelae?

**DOI:** 10.3390/jcm12062270

**Published:** 2023-03-15

**Authors:** Shahram Oliaei, Parinaz Paranjkhoo, SeyedAhmad SeyedAlinaghi, Esmaeil Mehraeen, Daniel Hackett

**Affiliations:** 1 HBOT Research Center, Golestan Hospital, Airspace and Diving Medicine Faculty, Navy and AJA Medical University, Tehran 166861955, Iran; 2 Turpanjian College of Health Sciences, American University of Armenia, Yerevan 0019, Armenia; 3 Iranian Research Center for HIV/AIDS, Iranian Institute for Reduction of High Risk Behaviors, Tehran University of Medical Sciences, Tehran 1419733141, Iran; 4 Department of Health Information Technology, Khalkhal University of Medical Sciences, Khalkhal 5681761351, Iran; 5 Physical Activity, Lifestyle, Ageing and Wellbeing Faculty Research Group, School of Health Sciences, Faculty of Medicine and Health, The University of Sydney, Sydney, NSW 2006, Australia

The COVID-19 pandemic has plagued our society for approximately three years. Over this period, multiple variants of the SARS-CoV-2 virus have been isolated and described, and various clinical challenges have been faced [1]. Consequently, there has been a continuous, evidence-based adaptation of existing COVID-19 guidelines and therapeutic recommendations [2]. In spite of these changes, oxygen therapy has remained a cornerstone for treating respiratory insufficiency in COVID-19 patients [3,4]. Indeed, the National Institute of Health guidelines recommend high-flow nasal cannula oxygen as a first-choice treatment in COVID-19 patients with hypoxemic respiratory failure [5].

Hyperbaric oxygen therapy (HBOT) has been proposed as an effective treatment for the reversal of acute complications and the management of patients with long-term symptoms of COVID-19 [4,6]. Despite the use of HBOT being absent from existing COVID-19 guidelines, recent evidence shows positive outcomes for its use in high-risk COVID-19 patients with refractory impairment of their respiratory function [7,8,9]. Interestingly, Robbins et al. reported that HBOT in ten patients with long complications of COVID-19 (i.e., refractory fatigue and perceived cognitive impairment) reduced fatigue and led to improvements in global cognition, attention, speech, and information processing [10]. This finding is consistent with a recent case report by Bhaiyat et al., where patients with long COVID treated with HBOT experienced significant clinical improvements in cognitive ability and cardiopulmonary function [11]. Importantly, the *British Medical Journal* recently published a protocol paper for a randomized, placebo-controlled, double-blind, parallel-groups clinical trial to evaluate the safety and efficacy of HBOT in patients with long COVID [12].

Due to the time that has elapsed since the emergence of COVID-19, it is likely that more cases of long-term complications will appear. Therefore, investigating effective treatment options for these complications is a research priority. As discussed in this letter, HBOT may be one of these effective methods for long COVID based on the growing clinical, epidemiological, as well as pathophysiological evidence. If future research corroborates the existing evidence on HBOT in long COVID, this therapy would certainly be a practical solution to a very impactful problem. Furthermore, the potential role of HBOT in the treatment of long COVID sequelae paves the way for an exciting therapeutic field and an additional weapon in the fight against the COVID-19 pandemic.
